# How Do *Trichoderma* Genus Fungi Win a Nutritional Competition Battle against Soft Fruit Pathogens? A Report on Niche Overlap Nutritional Potentiates

**DOI:** 10.3390/ijms21124235

**Published:** 2020-06-14

**Authors:** Karolina Oszust, Justyna Cybulska, Magdalena Frąc

**Affiliations:** Institute of Agrophysics, Polish Academy of Sciences, Doświadczalna 4, 20-290 Lublin, Poland; j.cybulska@ipan.lublin.pl (J.C.); m.frac@ipan.lublin.pl (M.F.)

**Keywords:** phytopathogens, beneficial fungi, nutrition competitiveness

## Abstract

We present a case study report into nutritional competition between *Trichoderma* spp. isolated from wild raspberries and fungal phytopathogenic isolates (*Colletotrichum* sp., *Botrytis* sp., *Verticillium* sp. and *Phytophthora* sp.), which infect soft fruit ecological plantations. The competition was evaluated on the basis of nutritional potentiates. Namely, these were consumption and growth, calculated on the basis of substrate utilization located on Biolog^®^ Filamentous Fungi (FF) plates. The niche size, total niche overlap and *Trichoderma* spp. competitiveness indices along with the occurrence of a stressful metabolic situation towards substrates highlighted the unfolding step-by-step approach. Therefore, the *Trichoderma* spp. and pathogen niche characteristics were provided. As a result, the substrates in the presence of which *Trichoderma* spp. nutritionally outcompete pathogens were denoted. These were adonitol, D-arabitol, i-erythritol, glycerol, D-mannitol and D-sorbitol. These substrates may serve as additives in biopreparations of *Trichoderma* spp. dedicated to plantations contaminated by phytopathogens of the genera *Colletotrichum* sp., *Botrytis* sp., *Verticillium* sp. and *Phytophthora* sp.

## 1. Introduction

Fungi are the dominant components of most terrestrial ecosystems [1]. However, there is one great concern that has been widely highlighted, which is the prevalence of plant fungal diseases. Fungi may attack both plants and fruit, thereby contributing to the unpredictable spoilage of agriproducts [2,3,4]. The crucial plant pathogens are those from the fungal genera *Colletotrichum, Botrytis* as well as *Verticillium* and fungal-like *Phytophthora.* These pathogens may wreak havoc, especially in organic production [5]. 

This is an important matter, since organic soft fruit production has been increasing constantly in recent years and has also enlarged its market share in worldwide food production [6]. Strawberry, raspberry and blueberry fruits are often included as crucial products of central Europe, with a seemingly endless increase in consumer demand to introduce organic methods of fruit cultivation. This constitutes a major reason to seek alternative ways to reduce the losses caused by pathogenic fungi [7]. 

In particular, *Colletotrichum* sp. and *Botrytis* sp., the causal actors of anthracnose and gray mould, respectively, require effective management strategies to combat them [8]. The *Verticillium* genus representatives, which cause wilt, are also critical from an economic point of view [9,10]. *Phytophthora* species are responsible for many losses in all of the production areas of the world [11]. 

Notably, the dynamic of ecological communities is not only rather menacingly shaped by pathogens but also by beneficial microorganisms [12]. Beneficial species share host plants with an array of pathogenic fungi that also inhabit the plant’s internal tissues. Therefore, interactions between these fungi are likely to occur on multiple temporal and spatial scales. Beneficial fungi occurrence in plants may alter disease symptoms to a significant extent [13,14]. 

In recent times, the process of the interaction between *Trichoderma* spp., pathogen and plant in securing pre-harvest organic soft fruit production was summarized and highlighted [15]. *Trichoderma* spp. fungi function as biocontrol agents and manifest a few common mechanisms. In fact, there are five main responses involved in attacking other fungi and promoting plant growth, these have been summarized recently [7]. They are, the production of inhibitory compounds, mycoparasitism, the inactivation of pathogen enzymes, induced resistance and finally, but not the least important mechanism, providing competition for nutrients and therefore for living space by forming mycelium biomass. 

Nutritional competition is one of the most common biological control activities. What is more, this property is very useful for plant protection [16]. Fungi belonging to the *Trichoderma* genus are widely known for very rapid growth and are regarded as aggressive competitors. They quickly colonize substrates and exclude slower growing pathogens. Recent studies have also explored the meaning of the endophytic activities of *Trichoderma* spp. for the welfare of plants [17]. 

In terms of ecosystem function, the survival opportunity of fungal pathogens and beneficial strains in a given environment depends on their ability to tolerate nutritional conditions within a stress-like competition [1].

The niche overlapping concept that pertains to competition for substrates, posits that niches are defined by the requirements and impacts of the species that are occupying those niches. This, in turn, determines whether a given set of species can coexist in a given ecological community and therefore provides a prospective tool for biocontrol [18]. Researchers have only recently begun to investigate the relevance of phenotypic heterogeneity for the competitive success of microorganisms in different natural scenarios. [19]. This is in agreement with the principle of competitive exclusion (limiting similarity) [20].

The nutritional niche of fungi with the Phenotype Microarray (PM), among others that use Biolog^®^ Filamentous Fungi (FF) plates, was examined previously [18]. An array of substrates on a microtiter plate was used to assess the exoenzymatic capacity of the tested fungi, since it is impossible to accurately mimic the in chemical environment of the plant inside the plant host. The method of PM that emerged has been proven to have a realistic potential of providing a high throughput of information about the phenotypes of microbial isolates [21,22,23,24]. 

Thus, the phenotypic consumption response is taken into consideration in the PM approach, namely how the cells respond colorimetrically to the nutritional conditions. This potentiates the results from respiration activity, which accompanies catabolic activity and is monitored at 490 nm [25,26]. On the other hand, the phenotypic turbidity response explaining the increase in microbial cell biomass formation as a reaction to nutritional conditions is also used. This potentiate is recorded as a change in the optical density at 750 nm [27,28,29]. The more intensive the colour formation in the PM method, the better the organism is able to nutritionally use (to consume or to grow on) the provided substrate. 

Nevertheless, as it was recently proved that fungal biomass can, relatively speaking, be developed without consuming too much substrate [30]. This was then explored in, e.g., the evaluation of the phytochemicals of apple pomace as prospective bio-fungicide agents against mycotoxigenic fungi [31]. However, this aspect, to the best of our knowledge, has not been previously taken into consideration within competition evaluation. We regard this approach as a future tool that may provide a wider insight into the probability of biocontrol effectiveness upon niche overlapping phenomena.

Therefore, we hypothesized that substrates for nutritional growth as well as nutritional consumption potentiates obtained using the PM method, based on Biolog FF plates^®^, will be diversified between *Trichoderma* spp. and soft fruit pathogens: *Colletotrichum* sp., *Botrytis* sp., *Verticillium* sp. and *Phytophthora* sp. We aimed to characterize nutritional niches in order to assess the probability of niche colonization by these fungi. We intended to give the example of strawberry fruit cell walls and emphasize the substrates in the presence of which *Trichoderma* spp. is more nutritionally competitive. 

## 2. Results

**Figure 1:**
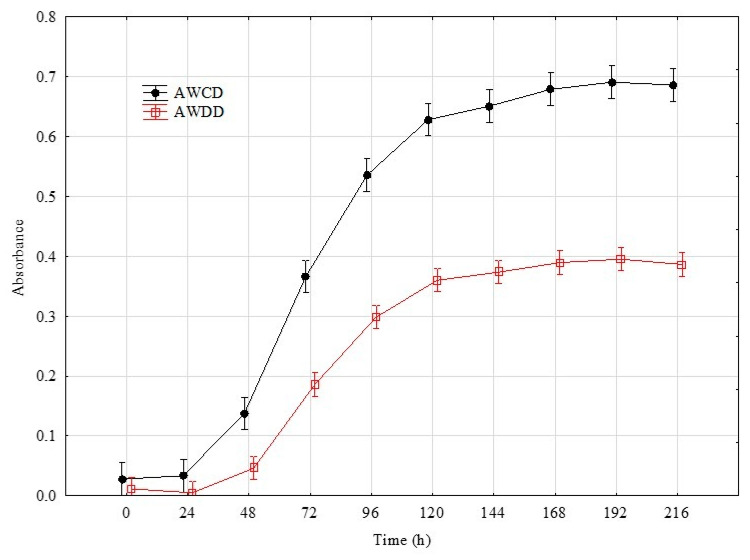
Changes in total AWCD and AWDD values calculated on the basis of the pathogenic fungi and beneficial *Trichoderma* isolates’ response to Filamentous Fungi (FF) Biolog^®^ substrates. Definitions: AWCD—Average Well Colour Development (A_490nm_), AWDD—Average Well Density Development (A_750nm_) (*n* = 3).

**Figure 2:**
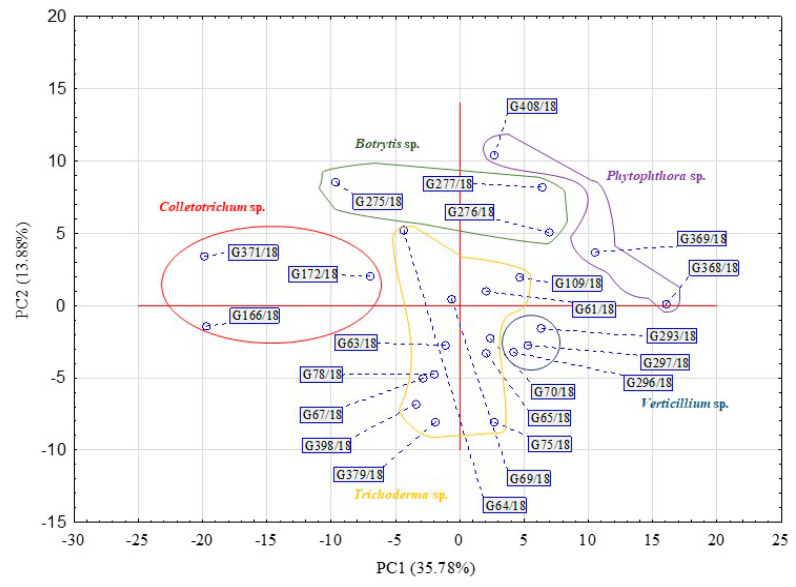
Principal component analysis (PCA) calculated on the basis of the pathogenic fungi and beneficial *Trichoderma* isolates’ response to FF Biolog^®^ substrates at 192 h (*n* = 3).

The rates in the total Average Well Colour Development (AWCD) and Average Well Density Development (AWDD) indices values were used to identify the time point that represents the greatest response of the set of tested fungal isolates. This approach was according to their consumption of different substrates and growth response, respectively (Figure 1). For further analyses, the time point of 192 h was taken into consideration with an average AWCD index value 0.7 and 0.4 for AWDD for the whole experiment data set. Principal component analysis (PCA) on the data of 192 h confirmed that the individual isolates belonging to the same genus clustered with respect to the isolate’s ability to consume and/or grow on particular sources (Figure 2).

Table 1 presents the rotated factor loadings and highly influential principal components (PC1: 35.78% and PC2 13.88%). There were 38 substrates, from the 95 available on an FF plate, which strongly and positively influence PC1 or PC2 for a fungal dataset. For the most part, if a substrate influences principal components, this influence originated from both consumption and growth potentiates. Only a minor difference was noted in the factors value obtained between the consumption and growth potentials. For example, it was for 2-amino ethanol that these were 0.807 and 0.779, respectively, influencing PC1 or for adenosine 0.825 and 0.719, respectively, influencing PC2. 

For some substrates, there were higher factor values noted for those represented by growth potentiates (putrescine, L-alanine, L-asparagine, L-serine, L-threonine, γ-amino-butyric acid, lactulose, succinic acid mono-methyl ester, D-ribose, maltitol, D-glucuronic acid). Moreover, exclusively, the consumption potentiates of such substrates as D-trehalose from the glucosides group, L-phenylalanine from L-amino acids, D-sorbitol from polyols and the growth potentiates of proline from L-amino acids, p-hydroxyphenyl acetic acid from other groups, fumaric acid belonging to the Tricarboxylic Acid (TCA) cycle-intermediates, have influenced principal components with no influence noted coming opposite from the growth and consumption of these substrates, respectively. This clearly suggests that both consumption and growth potentiates matter to a significant extent when evaluating fungi for nutritional differences that eventually make up the competition features.

The niche size, based on the total number of substrates used for the consumption and/or growth of the fungi of interest, varied among *Trichoderma* spp. and the genera group of specific pathogens (*Botrytis* sp., *Colletotrichum* sp., *Phytophthora* sp., *Verticillium* sp.) (Table 2). Nevertheless, the substrates that are most ubiquitously used by all of the tested fungi were found to belong to the oligosaccharides and peptides groups, when niche size evaluating. The most comparable niche size for the *Trichoderma* spp. among all of the tested pathogenic fungi was revealed to be *Colletotrichum* sp. *Phytophthora* sp. was noted to have the lowest niche size, being able to consume and grow on a low number of substrates. *Trichoderma* spp. was found to be superior to all other pathogens consuming 100% and growing on 88% of available hexoses; consuming 100% and growing on 25% of available aliphatic organic acids; consuming 75% of hexosamines and growing on 80% of pentoses. *Botrytis* sp. was found to have the greatest niche size among all tested fungi, when it comes to consuming 100% polysaccharides. *Colletotrichum* sp. and *Verticillium* sp. were found to most easily consume and grow on biogenic and heterocyclic amines (75% and 50%, respectively).

**Figure 3:**
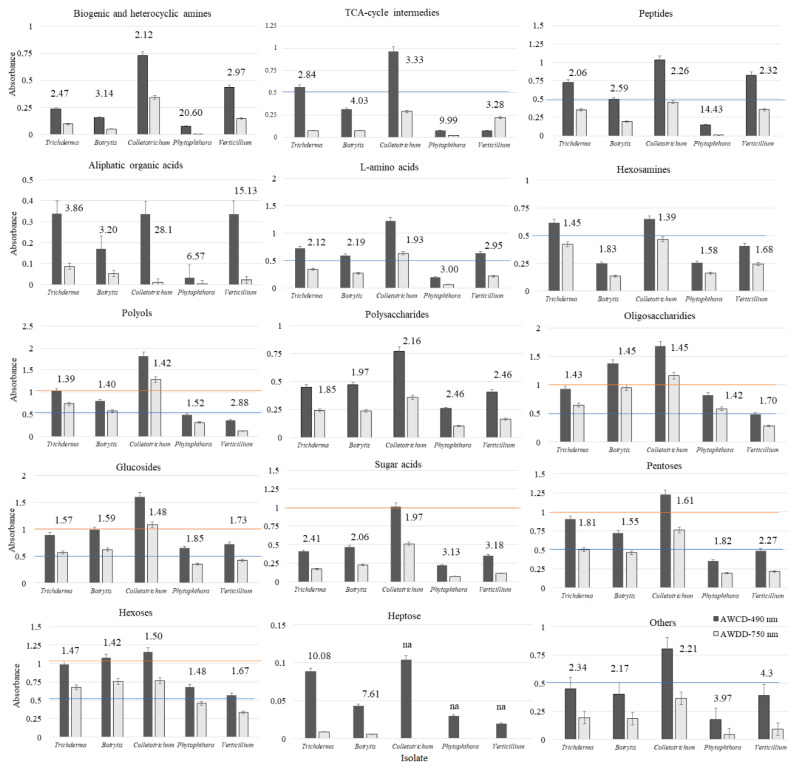
AWCD and AWDD ratio values of substrate groups calculated on the basis of consumption (Average Well Colour Development—AWCD, 490 nm) and growth (Average Well Density Development—AWDD) potentiates (A > 0.2, *n* = 3); “na” – indicates not available.

However, if the average nutritional response is analysed, namely the AWCD and AWDD values of the tested groups of substrates (Figure 3), it did not exactly match the findings noted for niche size. The greatest nutritional response (AWCD ≥1.0 and AWDD ≥0.5) among all of the tested fungi was met for such a group of substrates as polyols, oligosaccharides, glucosides, pentoses, hexoses. However, the lowest response (AWCD and AWDD ≤0.5) was for biogenic and heterocyclic amines, aliphatic and heterocyclic amines, polysaccharides, sugar acids, and heptose. The medium response (AWCD 0.5–1.0) was encountered on TCA-cycle intermediates, peptides, L-amino acids, hexosamines, and other groups. Nevertheless, for most substrate groups, at least one pathogen exceeded the response of *Trichoderma* spp. This was encountered for *Colletotrichum* sp. on polyols, glucosides and pentoses, and for *Colletotrichum* sp. and *Botrytis* sp. on oligosaccharides and hexoses. There was a trend met that *Colletotrichum* sp. dominates over *Trichoderma* spp. and other pathogens nutritionally, revealing greater catabolism and/or growth. *Trichoderma* spp. can match nutritionally *Colletotrichum* sp. on hexosamines intermediates. The average nutritional response was almost the same.

What is more, a stressful metabolic situation, indicated by the ratio of both AWCD to AWDD (Figure 3) was met when using L-amino acids, sugar acids and others for *Phytophthora* sp. and *Verticillium* sp. Stressful situation was noted on TCA-cycle intermediates and biogenic and heterocyclic amines for all tested pathogens, but not *Trichoderma* spp. Peptides caused metabolic stress only for *Phytophthora* sp., heptose for *Trichoderma* spp. and *Botrytis* sp., whereas aliphatic organic acids caused metabolic stress for *Trichoderma* spp. and all pathogens.

The NOI_TOT_ index (Table 3) was found to reach 1 mainly for *Colletotrichum* sp. due to polyols, L-amino acids, TCA- cycle intermediates consumption as was the case for growth polysaccharides, biogenic and heterocyclic amine, glucosides and polyols. As for *Botrytis* sp. and *Verticillium* sp. and the growth for these genera, the greatest NOI_TOT_ for polysaccharides and biogenic and heterocyclic amines, respectively was revealed. It was also confirmed that the most versatile substrates for all tested fungi belonged to oligosaccharides and peptides.

The COM_TRICH_ index (Table 3) reached >2.0, giving competitive consumption and growth dominance over *Botrytis* sp., on hexosamines. For *Phytophthora* sp., this phenomenon was observed on hexosamines and L-amino-acids. Consumption dominance over *Botrytis* sp. was met on aliphatic and organic acids. Growth dominance over *Botrytis* sp. was met on peptides and other groups, over *Phytophthora* sp. on pentoses and over *Verticillium* sp. on polysaccharides, glucosides, polyols and pentoses. 

It should be mentioned that high COM_TRICH_ index values were also noted, thereby explaining the growth dominance over *Colletotrichum* sp. and *Verticillium* sp. on biogenic and heterocyclic amines. However, simultaneously for this substrate group, a very low COM_TRICH_ index value was encountered, indicating the consumption superiority of those two pathogens over *Trichoderma* spp. 

*Trichoderma* spp. was able to produce a higher response than *Phytophthora* sp. on L-aminoamides, polysaccharides, sugar acids and others. *Phytophthora* sp. and *Botrytis* sp. reacted greatly on biogenic and heterocyclic amines and peptides. *Phytophthora* sp. and *Verticillium* sp. responded well on oligosaccharides, glucosides and hexoses, whereas *Phytophthora* sp., *Verticillium* sp. and *Botrytis* sp. on TCA-cycle intermediates, hexosamines, polyols and pentoses.

**Figure 4:**
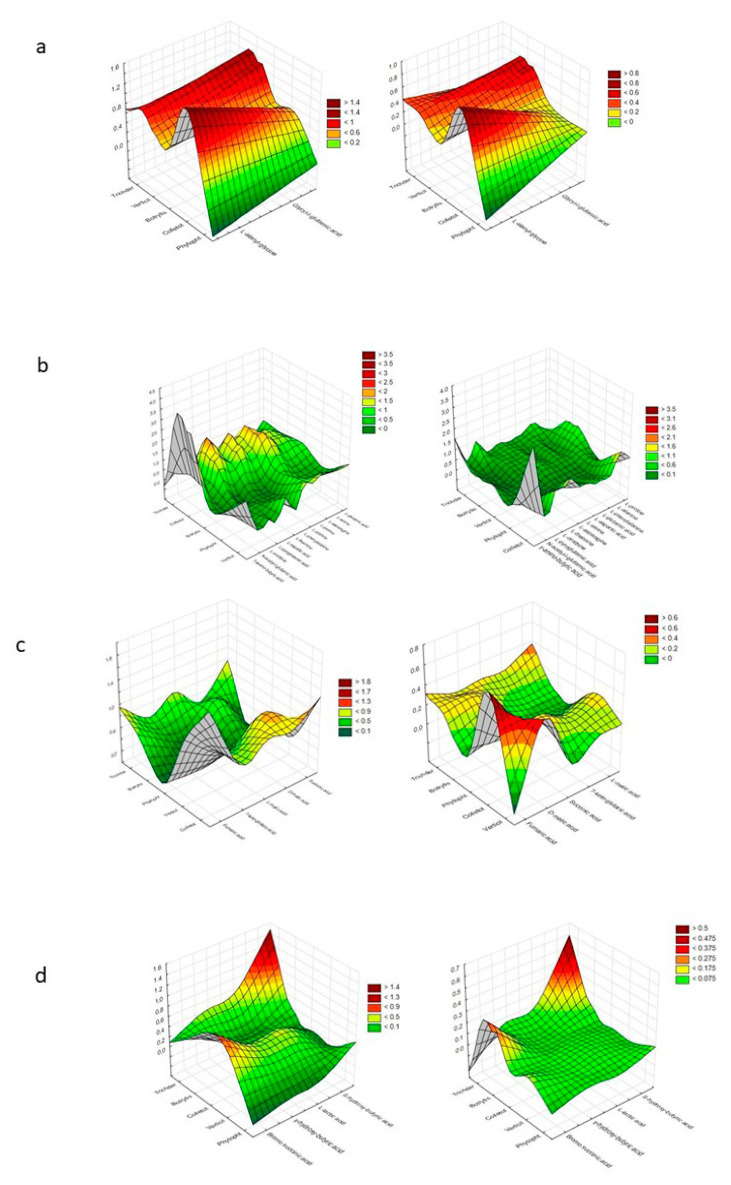

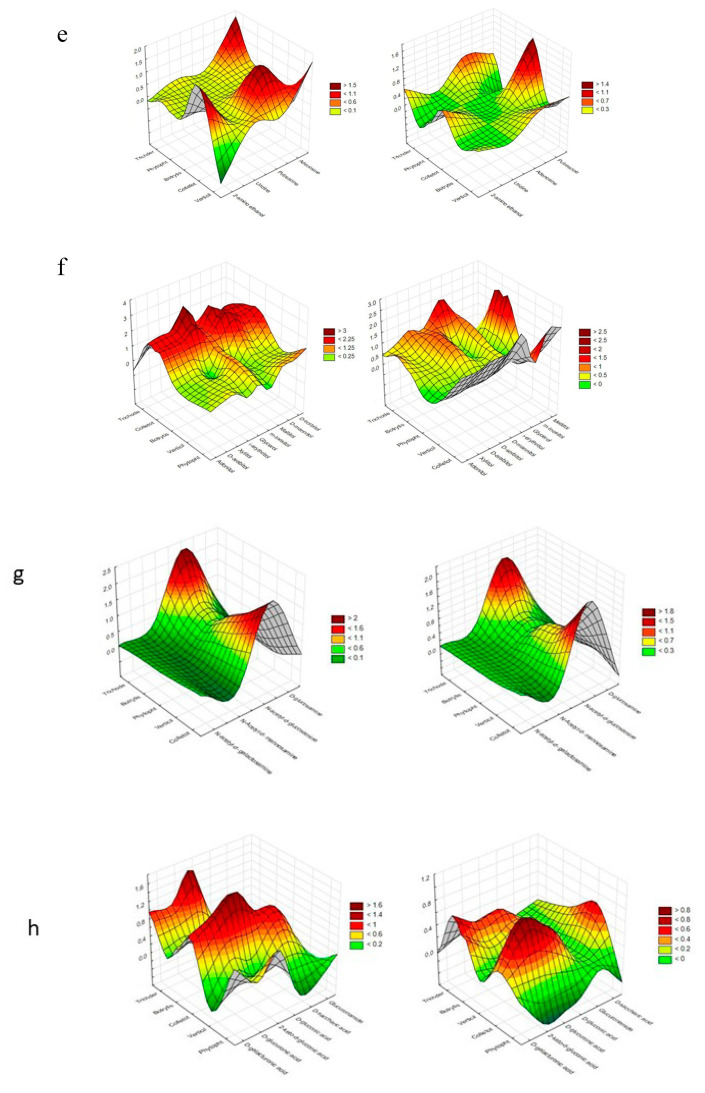

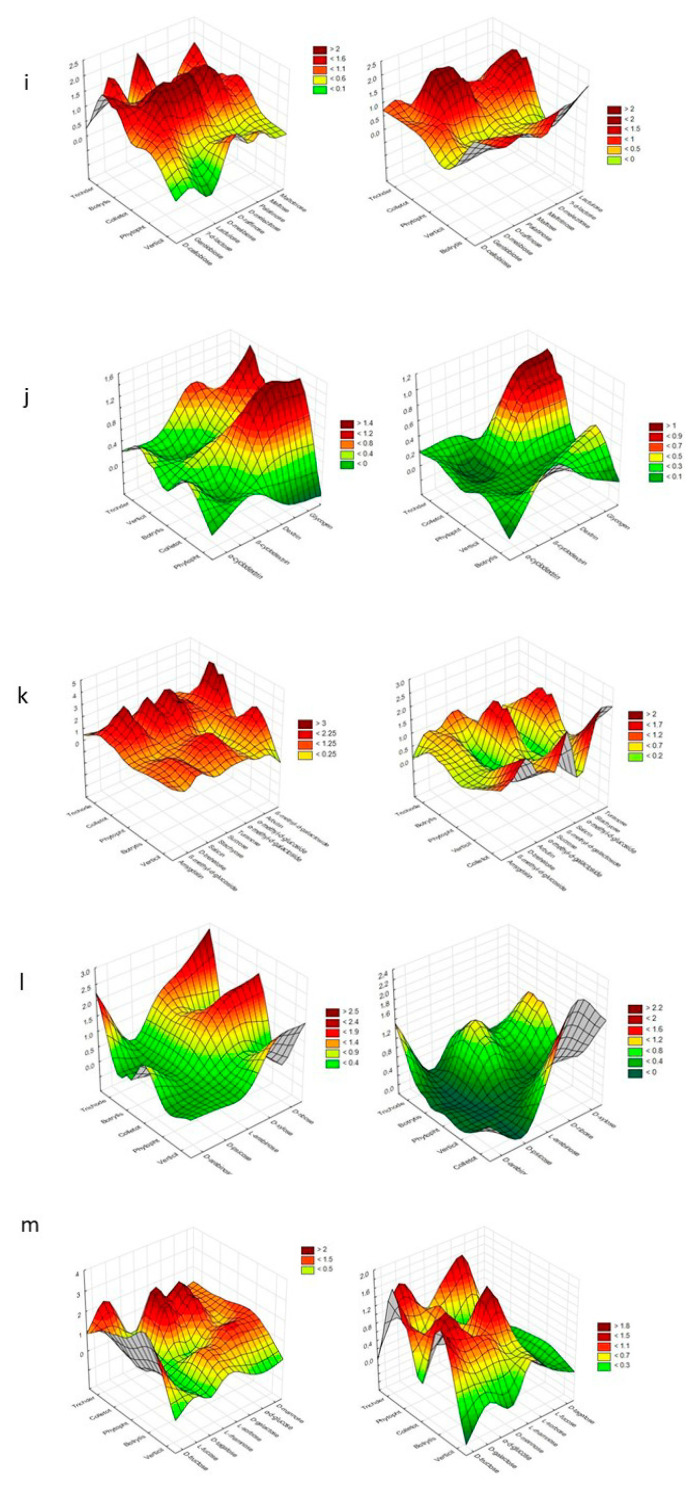
Cluster Analysis depicting the consumption response (A 490 nm, shown at left) and growth response (A 750 nm, shown at right) of microorganisms to substrates located on Biolog^®^ FF plates, shown as the following substrate groups: (**a**) peptides, **(b**) L-amino acids, (**c**) Tricarboxylic Acid (TCA) cycle-intermediates, (**d**) aliphatic organic acids, (**e**) biogenic and heterocyclic amines, (**f**) polyols, (**g**) hexosamines, (**h**) sugar acids, (**i**) oligosaccharides, (**j**) polysaccharides, (**k**) glucosides, (**l**) pentoses, and (**m**) hexoses. The analysis was performed on the basis of consumption (490 nm) and growth potentiates (A > 0.2, *n* = 3).

Figure 4 presents a denotation of preferred and non-preferred particular substrates among each group (those groups that provoke a stressful metabolic situation for *Trichoderma* spp. were excluded). 

It was assumed that mainly *Trichoderma* spp. and *Colletotrichum* sp. preferred the same particular substrates. These were as follows, from the peptides—L-alanyl-glycine and glycyl-l-glutamic acid; from L-amino acids—γ-amino-butyric acid; from TCA-cycle intermediates— fumaric acid; from hexosamines—N-acetyl-D-glucosamine. 

Polysaccharides such as dextrin and glycogen, hexoses such as D-fructose, D-galactose, α-D-glucose, D-mannose, L-rhamnose, D-tagatose, and L-sorbose, sugar acids such as D-galacturonic acid, D-gluconic acid, D-glucuronic acid, 2-keto-D-gluconic acid, and D-saccharic acid, polyols such as adonitol, D-arabitol, i-erythritol, glycerol, D-mannitol, D-sorbitol, xylitol, inositol, and maltitol, and biogenic and heterocyclic amines such as 2-amino ethanol and putrescine were even more preferred by *Colletotrichum* sp. than *Trichoderma* spp. 

*Trichoderma* spp. preferred the polyols adonitol, D-arabitol, i-erythritol, glycerol, D-mannitol, and D-sorbitol, and, from biogenic and heterocyclic amines, adenosine.

Pentoses such as D-arabinose, L-arabinose, D-ribose, and D-xylose were preferred to a high degree by *Trichoderma* spp., *Colletotrichum* sp. and *Verticillium* sp. 

Glucosides—amygdalin, arbutin, α-methyl-D-galactoside, *β*-methyl-D-glucoside, salicin, stachyose, sucrose, D-trehalose, and turanose—were preferred to an equal extent by *Trichoderma* spp. and *Botritis* sp.

Table 4 presents the saccharide composition of cell wall material extracted from strawberries cv. *Dipret* from organic farming. Sugar acids (galacturonic acid), pentoses (arabinose, xylose) and hexoses (rhamnose, galactose, glucose, mannose) were measured. Galacturonic acid (47.9 mol%), glucose (24.2 mol%), arabinose (12.1 mol%), and galactose (9.1 mol%) were revealed to be the most abundant. A low content of xylose (1.8 mol%), rhamnose (3.0 mol%) and mannose (1.9 mol%) was determined. 

## 3. Discussion

Nutritional potentiates were previously reported to be useful in niche overlap evaluation following the phenotype Microarray approach and based on substrate consumption. This was applied to Dutch elm fungal endophytes and pathogens [18]. 

As for the microorganisms of interest in soft fruit plantation, global substrate assimilation within mycelial growth was previously assessed and described as the metabolic profiles characteristic of *Trichoderma* spp. strains [32], *Phytophthora* sp. and *Botrytis* sp. [23]. Moreover, to the best of our knowledge, *Verticillium* sp. and *Colletotrichum* sp. have not been tested in this way. An array of substrates on a microtiter plate was used to assess the exoenzymatic capacity of the tested fungi. 

Following our hypothesis of nutritional growth as well as nutritional consumption, potentiates obtained using the PM method, based on Biolog FF plates^®^, were noted to be diversified between *Trichoderma* spp. and *Colletotrichum* sp., *Botrytis* sp., *Verticillium* sp. and *Phytophthora* sp., and thus it has an important meaning in niche evaluation, which included the competition for substrate groups (niche size, niche overlap index, competitiveness), the stressful metabolic situation, and substrates usage selectivity. 

We regard this approach as a future tool for providing a wider insight into the probability of biocontrol effectiveness. Substrates, such as those preferred by *Trichoderma* spp., but not by pathogens, may be considered as additives to *Trichoderma* spp. biopreparations and are expected to increase their competitiveness in the destined microbial community, e.g., community of soil or plant tissue beset by pathogens. 

It was revealed that *Trichoderma* spp. has the most similar niche to *Colletotrichum* sp. and follows the limiting similarity principle (competitive exclusion), these two species cannot occupy the same ecological niche [33]. Therefore, additives’ conception with adenosine, revealed in this study, may bring about a positive effect, especially against *Colletotrichum* sp.

Apart from adenosine, adonitol, D-arabitol, i-erythritol, glycerol, D-mannitol, and D-sorbitol can also be added to *Trichoderma* spp. biopreparations dedicated to plantations, where *Colletotrichum* sp., *Botrytis* sp., *Verticillium* sp. and *Phytophthora* sp. appear. Nevertheless, there are few substrates denoted that are preferred not only by *Trichoderma* spp. but also by particular pathogens, and, therefore, these may be considered as additives but, interestingly, it depends on which plates the fungal infection occurs. For example, L-alanyl-glycine and glycyl-l-glutamic acid, γ-amino-butyric acid, fumaric acid, N-acetyl-d-glucosamine could be less effective as additives applied, if *Colletotrichum* sp. occurred, but not for *Verticillium* sp., *Phytophthora* sp. and *Botrytis* sp. 

Our studies showed that, in particular, hexosamines groups are expected to increase *Trichoderma* spp. competitiveness against *Botrytis* sp. and *Phytophthora* sp. Oligosaccharides and peptides groups were the most ubiquitously used by all of the fungi tested and therefore would probably not give *Trichoderma* spp. much predominance in the community. 

The nutritional advantage of *Trichoderma* spp. also results from the fact that this group of fungi demonstrated a relatively low metabolic stress situation in the presence of only a few substrate groups compared to the pathogens. The above fact leads to the premise that *Trichoderma* spp. has a vast ability to develop in the environment. This beneficial aspect is the most desired activity in a fungal community and indicates the ability of *Trichoderma* spp. to more effectively colonize numerous and various niches [34]. Colonization is the very first step in the use of a wide array of other biological control mechanisms, such as antibiosis, antagonism, mycoparasitism, and the induction of plant defence responses [35]. 

Moreover, our findings related to strawberry fruit saccharides composition and the determination of preferred and non-preferred particular substrates among saccharides (glucose, mannose, rhamnose, galacturonic acid, arabinose) indicate that saccharide composition may be one of many conditions that results in *Colletotrichum* sp. and *Verticillium* sp. colonization and consequently in anthracnose and Verticillium wilt diseases development. It seems that in *Phytophthora* sp. and *Botrytis* sp. colonization, the other substrates play a crucial role. However, it is worth noting that, according to the results obtained, *Botrytis* sp. intensively utilizes galacturonic acid, which is one of the main components of strawberry, which may explain why these pathogens develop so easily on strawberry fruit, causing them to spoil. Nevertheless, in the community, a suite of traits is selected, which also maximizes the ability of the organism to acquire limiting resources given local environmental conditions in competition with co-occurring species [36]. 

In summary, to respond to the question of what makes *Trichoderma* spp. win the competition battle over the pathogens of soft fruits, the metabolic studies of beneficial *Trichoderma* spp. strains mainly included the determination of the food competition between these fungi, isolated from the rhizosphere and rhizoplane of wild raspberries, and phytopathogens (*Colletotrichum* sp., *Botrytis* sp., *Verticillium* sp., *Phytophthora* sp.) attacking the organic plantations of soft fruit. Based on the research conducted, it may be concluded that the substrates preferred by *Trichoderma* spp., but not by pathogens, can be used as additives for biopreparations containing these beneficial fungi. 

The results indicate that adenosine enhanced the growth of *Trichoderma* spp., but it was a source not utilized by *Colletotrichum* sp. fungi. These findings suggest that the addition of adenosine to biopreparations containing *Trichoderma* spp. may simultaneously stimulate beneficial fungi growth and negatively affect the phytopathogens of *Colletotrichum* sp. It has also been shown that adonitol, D-arabitol, i-erythritol, glycerol, D-mannitol and D-sorbitol can be added to the biopreparations of *Trichoderma* spp., and dedicated to plantations contaminated by phytopathogens of the genera *Colletotrichum* sp., *Botrytis* sp., *Verticillium* sp. and *Phytophthora* sp.

## 4. Materials and Methods 

### 4.1. Fungal Strains

The following fungal pathogens were used in the study and were isolated in the Laboratory of Molecular and Environmental Microbiology, Institute of Agrophysics, Polish Academy of Sciences: two strains of *Colletotrichum* sp. G166/18 (GenBank: MT126798.1), G172/18 (GenBank: MT126803.1)) were isolated from infected strawberry fruit. Three strains of *Botrytis* sp. G277/18 (GenBank: MT154304.1), G275/18 (GenBank: MT154302.1), G276/18 (GenBank: MT154303.1) and three strains of *Verticillium* sp. G293/18 (GenBank: MT133324.1), G296/18 (GenBank: MT133320.1), G297/18 (GenBank: MT133316.1) and one strain of *Phytophthora* sp. G408/18 (GenBank: MT126670.1) were isolated from infected strawberry roots. The two environmental strains of *Phytophthora* sp. (G368/18 (GenBank: MT558571), G369/18 (GenBank: MT558729)) and one strain of *Colletotrichum* sp. (G371/18 (GenBank: MT558572)) came from the collection of the Research Institute of Horticulture in Skierniewice (Poland).

Twelve isolates of *Trichoderma* spp. were used: G109/18, G61/18, G65/18, G67/18, G69/18, G70/18. They were isolated in the Laboratory of Molecular and Environmental Microbiology, Institute of Agrophysics, Polish Academy of Sciences from wild raspberry rhizosphere soil using a serial dilution method; G379/18 and G398/18 were isolated from the external surface roots together with closely adhering soil particles and debris (rhizoplane); and G75/18 (GenBank: MT558563), G63/18 (GenBank: MT558561), G64/18 (GenBank: MT558562), G78/18 were isolated from wild raspberry roots.

All environmental samples used to isolate the fungi were obtained from Poland. Strawberry/raspberry roots were washed in tap water (for a few minutes in a bowl), then thoroughly rinsed with distilled water, then a surface disinfection in 70% ethanol was performed. Then the top layer of the root was removed and the interior part was cut into small fragments (several mm) and laid on a prepared media in Petri dishes (Potato Dextrose Agar, PDA, Biocorp, Warsaw, Poland). The serial dilutions method was used to isolate fungi from the rhizosphere and rhizoplane. In order to obtain the growth of the microbes, incubation was conducted at 22 °C. The passage of the fresh PDA medium was conducted several times to obtain a pure culture of fungi. 

Fruit with visible traces of infestation (black lesions) were cut without the removal of changed fruit fragments and placed in a sterile medium (V8 or PDA, Biocorp, Warsaw, Poland). Incubation at 22 °C for several days (until the mycelium appears) allowed for the isolation of the fungi. The passage of the fresh PDA medium was conducted several times to obtain a pure culture of all the strains. 

Genetic genus identification was confirmed using Internal Transcribed Spacer (ITS) region [37] or/and D2 Region of the Large Subunit Ribosomal RNA Gene (D2 LSU) [38] gene fragments as described by Frąc et al. [39].

All of the analyses were performed using mean values for the data obtained from the isolates mentioned above (with three independent replicates) divided according to the distinction of being a member of the following groups: *Trichoderma* spp., *Botrytis* sp., *Colletotrichum* sp., *Phytophthora* sp., *Verticillium* sp. 

### 4.2. FF Plates^®^ Preparation

The inoculation procedure was performed according to the manufacturer’s protocol with modifications as described in detail by [21] in three replicates (three separate plates for each isolate). In brief, after the homogenization of the mycelium suspension in inoculating fluid (FF-IF, Biolog^®^, Hayward, CA, USA), the transmittance was adjusted to 75% using a turbidimeter (Biolog^®^). A volume of 100 μL of the mycelium suspension was added to each well. The inoculated microplates were incubated in darkness at 25 °C within 10 days. 

### 4.3. Group of Substrate Use—Specific Phenotypic Profiles Based on Consumption and Growth Potentiates

The optical density at 490 nm (substrate consumption, catabolism, respiration) and 750 nm (turbidity, growth, biomass formation) was determined using a microplate reader (Biolog^®^, Hayward, CA, USA) on a daily basis to calculate Average Well Colour Development (AWCD) and Average Well Density Development (AWDD) indices, respectively, as suggested by Jeszka-Skowron et al. [29]. 

Fifteen groups of these substrates were evaluated in accordance with [40] based on their chemical properties. These were as follows heptoses, hexoses, pentoses, sugar acids, hexosamines, polyols, polysaccharides, oligosaccharides, glucosides, peptides, L-amino acids, biogenic and heterocyclic amines, TCA-cycle intermediates, aliphatic organic acids, and others. For interpretation, the substrates were also divided into more general groups, namely into: monosaccharides (heptose, hexoses, pentoses), monosaccharides-related substrates (sugar acids, hexosamines, polyols), sugar-related substrates (polysaccharides, oligosaccharides), N-containing substrates (peptides, L-amino acids, biogene and heterocyclic amines, TCA-cycle intermediates, aliphatic organic acids) and others (glucosides) [30,41].

### 4.4. Time Point Selection

The rates of change in the total AWCD and AWDD indices values dynamics (mean for all tested strains) and the principal component analysis (PCA) were used to identify and confirm the time point (the reading hour) that best represents the greatest response of the fungal isolates according to their consumption of different substrates and growth responses, respectively. The varimax-rotated factor loadings substantially influence the principal components (>0.7) which were distinguished. An optical density higher than 0.20 for each substrate was considered to be a positive response.

### 4.5. Competition for Substrates Groups

In order to compare the substrate group use patterns of the pathogens to those of the beneficial *Trichoderma* spp., AWCD and AWDD indices, niche size, a total niche overlap index (NOI_TOT_) and *Trichoderma* spp. competitiveness index (COM_TRICH_) were calculated according to [18] with their own modification. In brief, the niche size is the share of the positive response (%) to the substrate groups. 

The NOI_TOT_ index was calculated as the number of substrates shared by both pathogens and *Trichoderma* spp. divided by the total number of substrates given in a particular group. The NOI_TOT_ value of 0.9 or above was assumed to indicate a high degree of niche overlap and a competitive advantage for the target fungus [18,42]. This function was used to quantify the ability of *Trichoderma* spp. to overcome pathogens. It was assumed that, if the value of this function was greater than 2.0, *Trichoderma* spp. exhibits a competitive superiority in relation to the pathogen.

The COM_TRICH_ value indicates the relative rate of substrate usage by the pathogenic isolates compared to *Trichoderma* spp. (calculated as *Trichoderma* spp. effectiveness at using substrates included in a particular group, in comparison to pathogens). A value of 2.0 or higher indicates that the *Trichoderma* spp. strain is more effective at utilizing the substrates included in a particular group. A value below 1.0 means that the pathogen is more successful at substrate usage.

### 4.6. Stressful Metabolic Situation

The ratio was calculated for AWCD and AWDD of the substrate group for each group of fungi to indicate the specific respiration rate for the mean values of each substrate group and shows the catabolic efforts, compared with biomass development. A ratio much higher than 2.0 was regarded as indicating a stressful metabolic situation, when a small biomass yields high respiration rates [30,41].

### 4.7. Substrate Usage Selectivity—Preferred and Non-Preferred Substrates

The cluster analysis, particularly the grouping of objects and features with superficies visualization, was performed to denote preferred and non-preferred particular substrates among a group.

### 4.8. Saccharide Composition of Cell Wall Material from Strawberries

The cell wall material was extracted from strawberries cv. *Dipret* purchased from a local organic farmer according to the method by Renard [43] with slight modifications. The strawberries were first homogenized and then mixed and stirred with 70% ethanol for 1 h. Next, the mixture was filtered and mixed repeatedly with ethanol until a negative result was obtained from the assay concerning the presence of sugars [44].

Saccharide composition was determined according to a modified method described by Lv et al. [45]. In brief, cell wall material from strawberries (CWM) was decomposed by hydrolysis in trifluoroacetic acid (TFA) by the addition of 2 mL of 3M TFA into a glass tube with 20 mg of CWM and incubation in boiling water for 8 h. After cooling, the suspension was centrifuged and the supernatant was freeze-dried and then 1 mL of water was added to the hydrolysate. 

Hydrolysed saccharides were subjected to derivatization with 1-Phenyl-3-methyl-5-pyrazolone (PMP). First, 50 μL of 0.3M NaOH and a 0.5 M methanol solution of PMP were added to hydrolysate. The mixture was incubated for 60 min at 70 °C and then cooled, neutralized with 0.3M HCl and extracted three times with 1 mL of chloroform. The aqueous layer was then filtered through a 0.45 μm membrane. 

The concentration of PMP-labelled saccharides was then determined using a High Performance Liquid Chromatography (HPLC) system equipped with a S 1130 HPLC quaternary pump, S 5300 sample injector, S4120 column oven and S 3350 PAD detector (Sykam GmbH, Gewerbering, Germany). The HPLC column was a Bionacom Velocity LPH C18 (ID 4.6 × 250 mm, 5 μm), preceded by a 0.5 µm Bionacom ultra filter column protector. The injection volume was 20 μL, the flow rate was 0.8 mL min^−1^ and the temperature was 35 °C. The chromatograms were recorded at 248 nm. Two mobile phases, A (acetonitrile) and B (0.045% KH_2_PO_4_–0.05% triethylamine buffer, pH 7.0), were applied with a gradient elution of 90–89–86% B with a linear decrease from 0–15–40 min. Saccharide concentration was then calculated on the basis of the calibration curves that were composed of five concentrations of PMP-labelled standards: galacturonic acid, arabinose, rhamnose, galactose, glucose, rhamnose, xylose, mannose and fucose. 

## 5. Conclusions

In summary, to respond to the question of what makes *Trichoderma* spp. gain supremacy in a competition battle with soft fruit pathogens, the metabolic studies of beneficial *Trichoderma* spp. strains mainly included the determination of food competition between these fungi, isolated from the rhizosphere and rhizoplane of wild raspberries, and phytopathogens (*Colletotrichum* sp., *Botrytis* sp., *Verticillium* sp., *Phytophthora* sp.) attacking organic plantations of soft fruit. Based on the research conducted, it may be concluded that the substrates, those preferred by *Trichoderma* spp., but not by pathogens, can be used as additives in biopreparations containing these beneficial fungi. The results indicate that adenosine enhanced the growth of *Trichoderma* spp., but it was a source that was not utilized by *Colletotrichum* sp. fungi.

This finding suggests that the addition of adenosine to biopreparations containing *Trichoderma* spp. can simultaneously stimulate beneficial fungi growth and can also negatively affect the phytopathogens of *Colletotrichum* sp. It has also been shown that adonitol, D-arabitol, i-erythritol, glycerol, D-mannitol and D-sorbitol can be added into the biopreparations of *Trichoderma* spp., and dedicated to plantations contaminated by phytopathogens of the genera *Colletotrichum* sp., *Botrytis* sp., *Verticillium* sp. and *Phytophthora* sp.

## Figures and Tables

**Table 1:** Rotated factor loadings with the principal components (PC) distinguished (PC1: 35.78% and PC2 13.88%), calculated on the basis of the pathogenic fungi and beneficial *Trichoderma* isolates response to FF Biolog^®^ substrates, namely the consumption (490 nm) and growth (750 nm) potentiates (A > 0.2, *n* = 3, 192 h); “-“ means lack of particular substrate group influence on PC; bold numbers mean its significant influence on PC (PC ≥ 0.7).

Substrate Group	Substrate	PC1	PC2	PC1	PC2
		490 nm		750 nm	
*Biogenic and heterocyclic amines*	2-amino ethanol	**0.807**	0.044	**0.779**	0.065
	Putrescine	**0.720**	0.111	**0.755**	0.051
	Adenosine	−0.517	**0.825**	−0.091	**0.719**
*Glucosides*	D-trehalose	**0.740**	0.073	-	-
	ß-methyl-D-glucoside	**0.828**	0.143	**0.744**	0.148
	Stachyose	**0.903**	−0.016	**0.874**	−0.109
*Hexoses*	D-galactose	**0.821**	−0.315	**0.785**	−0.341
	L-rhamnose	**0.774**	−0.143	**0.777**	−0.198
*L-amino acids*	L-alanine	**0.722**	−0.024	**0.771**	−0.043
	L-asparagine	**0.731**	0.248	**0.747**	0.156
	L-phenylalanine	0.394	**0.720**	-	-
	L-proline	-	-	**0.721**	−0.081
	L-serine	**0.758**	0.232	**0.766**	0.182
	L-threonine	**0.848**	0.356	**0.858**	0.272
	γ-amino-butyric Acid	**0.735**	0.445	**0.805**	0.245
*Oligosaccharides*	D-melibiose	**0.879**	−0.039	**0.852**	0.028
	D-raffinose	**0.817**	−0.171	**0.793**	−0.275
	Lactulose	**0.765**	−0.396	**0.687**	−0.433
	Palatinose	**0.748**	−0.487	**0.726**	−0.482
	α-D-lactose	**0.774**	−0.286	**0.711**	−0.306
*Others*	p-hydroxyphenyl acetic acid	-	-	**0.745**	0.111
	Quinic acid	**0.932**	0.032	**0.931**	0.003
	Succinic acid mono-methyl ester	**0.798**	0.355	**0.854**	0.172
*Pentoses*	D-ribose	**0.842**	0.344	**0.861**	0.268
	D-xylose	**0.880**	−0.157	**0.873**	−0.338
*Peptides*	L-alanyl-glycine	**0.765**	0.435	**0.757**	0.353
*Polyols*	Adonitol	**0.717**	0.324	**0.706**	0.328
	D-arabitol	**0.795**	0.166	**0.785**	0.113
	D-mannitol	**0.777**	0.044	**0.750**	0.018
	D-sorbitol	**0.744**	0.055	-	-
	Maltitol	**0.803**	−0.484	**0.788**	−0.506
	m-inositol	**0.820**	0.226	**0.793**	0.185
*Polysaccharides*	Dextrin	**0.907**	0.111	**0.788**	0.200
*Sugar acids*	2-keto-D-gluconic acid	**0.831**	0.166	**0.795**	0.147
	D-galacturonic acid	**0.816**	−0.149	**0.738**	−0.194
	D-glucuronic acid	**0.836**	0.205	**0.841**	0.155
*TCA-cycle intermediates*	Fumaric acid	-	-	**0.729**	0.365
	α-keto-glutaric acid	0.448	**0.734**	0.236	**0.789**

**Table 2:** The share of FF Biolog^®^ substrate group positive response (%) of the pathogenic fungi and beneficial *Trichoderma* strains, calculated on the basis of consumption (Average Well Colour Development—AWCD, 490 nm) and growth (Average Well Density Development—AWDD) potentiates (A > 0.2, *n* = 3).

Substrate Group	Utilization (%)									
	AWCD					AWDD				
	*Trichoderma*	*Botrytis*	*Colletotrichum*	*Phytophthora*	*Verticillium*	*Trichoderma*	*Botrytis*	*Colletotrichum*	*Phytophthora*	*Verticillium*
*Polysaccharides*	75	100	75	50	75	50	50	50	0	25
*Biogenic and heterocyclic amines*	25	25	75	25	75	25	0	50	0	50
*Glucosides*	82	63	82	73	55	73	64	82	64	18
*Polyols*	100	78	100	89	67	89	78	100	78	22
*Aliphatic organic acids*	100	25	75	0	75	25	0	0	0	0
*L-amino acids*	100	83	100	42	83	83	67	92	8	67
*TCA-cycle intermediates*	100	80	100	40	100	40	0	60	0	40
*Sugar acids*	83	83	83	50	50	50	67	83	0	33
*Heptoses*	0	0	0	0	0	0	0	0	0	0
*Oligosaccharides*	100	100	100	100	80	80	100	100	100	70
*Hexosamines*	75	25	50	25	50	50	25	50	25	50
*Hexoses*	100	75	88	75	88	88	75	75	63	75
*Pentoses*	80	80	80	60	60	80	60	60	40	40
*Peptides*	100	100	100	0	100	100	50	100	0	100
*Others*	80	80	80	30	70	50	20	50	0	30

**Table 3:** Total Niche Overlap Index (NOI_TOT_) and *Trichoderma* Competitiveness Index (COM_TRICH_). The presented indices were calculated on the basis of consumption (Average Well Colour Development—AWCD, 490 nm) and growth (Average Well Density Development—AWDD) potentiates (A > 0.2, *n* = 3).

*Substrate Group*	^a^ NOI_TOT_								^b^ COM_TRICH_							
	AWCD				AWDD				AWCD				AWDD			
	*Botrytis*	*Colletotrichum*	*Phytophthora*	*Verticillium*	*Botrytis*	*Colletotrichum*	*Phytophthora*	*Verticillium*	*Botrytis*	*Colletotrichum*	*Phytophthora*	*Verticillium*	*Botrytis*	*Colletotrichum*	*Phytophthora*	*Verticillium*
Polysaccharides	0.75	0.75	0.50	0.75	**1.00**	**1.00**	-	0.50	0.75	1.00	1.50	1.00	1.00	1.00	-	**2.00**
Biogenic and heterocyclic amines	0.25	0.25	0.25	0.25	-	**1.00**	-	**1.00**	1.00	0.33	1.00	0.33	-	**2.00**	-	**2.00**
Glucosides	0.64	0.82	0.73	0.55	0.88	**1.00**	0.88	0.25	1.29	1.00	1.13	1.50	1.14	0.89	1.14	**4.00**
Polyols	0.78	**1.00**	0.89	0.67	0.88	**1.00**	0.88	0.25	1.29	1.00	1.13	1.50	1.14	0.89	1.14	**4.00**
Aliphatic organic acids	0.25	0.75	-	0.75	-	-	-	-	**4.00**	1.33	-	1.33	-	-	-	-
L-amino acids	0.83	**1.00**	0.42	0.83	0.67	0.92	0.08	0.67	1.20	1.00	**2.40**	1.20	1.25	0.91	**10.00**	1.25
TCA-cycle intermediates	0.80	**1.00**	0.40	0.60	-	0.40	-	0.40	1.25	1.00	2.50	1.00	-	1.50	-	1.00
Sugar acids	0.83	0.83	0.50	0.50	0.50	0.50	-	0.33	1.00	1.00	1.67	1.67	0.75	0.80	-	1.50
Heptose	-	-	-	-	-	-	-	-	-	-	-	-	-	-	-	-
Oligosaccharides	**1.00**	**1.00**	**1.00**	0.80	0.80	0.80	0.80	0.70	1.00	1.00	1.00	1.25	0.80	0.80	0.80	1.14
Hexosamines	0.25	0.50	0.25	0.50	0.25	0.50	0.25	0.50	**3.00**	1.50	**3.00**	1.50	**2.00**	1.00	**2.00**	1.00
Hexoses	0.75	0.88	0.75	0.88	0.75	0.75	0.63	0.75	1.33	1.14	1.33	1.14	1.17	1.17	1.40	1.17
Pentoses	0.80	0.80	0.60	0.60	0.60	0.60	0.40	0.40	1.00	1.00	1.33	1.33	1.33	1.33	**2.00**	**2.00**
Peptides	**1.00**	**1.00**	-	**1.00**	0.50	**1.00**	-	**1.00**	1.00	1.00	-	1.00	**2.00**	1.00	-	1.00
Others	0.80	0.80	0.30	0.70	0.20	0.50	-	0.30	1.00	1.00	**2.67**	1.14	**2.50**	1.00	-	1.67

^a^ The niche overlap index compares the number of substrates used by both the pathogen and the endophyte to the total number of substrates that could be utilized. A value of 1 or higher (in bold) indicates a high degree of niche overlap. ^b^ *Trichoderma* competitiveness indicates the relative rate of substrate usage by the pathogen compared to *Trichoderma*. A value of 2 or higher (in bold) indicates that *Trichoderma* is more effective at utilizing the substrates included in a particular group. A value below 1 (indicated by underlining) pathogen is more successful at substrate usage. “–“ indicates no response.

**Table 4:** Saccharide composition of strawberry fruit (mol%).

Sugar Acid	Pentoses		Hexoses			
Galacturonic Acid	Arabinose	Xylose	Rhamnose	Galactose	Glucose	Mannose
47.9 ± 0.3	12.1 ± 0.2	1.8 ± 0.1	3.0 ± 0.2	9.1 ± 0.2	24.2 ± 0.2	1.9 ± 0.1

## Abbreviations

FF	Filamentous Fungi
COM_TRICH_	*Trichoderma* Competitiveness Index
NOI_TOT_	Total Niche Overlap index
AWCD	Average Well Colour Development
AWDD	Average Well Density Development
HPLC	High Performance Liquid Chromatography
PCA	Principal Component Analysis
PDA	Potato Dextrose Agar
TCA	Tricarboxylic Acid Cycle / Citric Acid Cycle
CWM	Cell Wall Material
TFA	Trifluoroacetic Acid
PMP	1-Phenyl-3-methyl-5-pyrazolone
FF-IF	Biolog^®^ Filamentous Fungi Inoculating Fluid
ITS	Internal Transcribed Spacer region
D2 LSU	D2 Region of the Large Subunit Ribosomal RNA Gene
